# Rare penetrating abdominal injury caused by falling from height: Miraculously good prognosis

**DOI:** 10.3389/fsurg.2022.1018003

**Published:** 2022-10-31

**Authors:** Yanquan Liu, Qinglin Xu, Hongquan Zhu, Jun Wang, Fanlin Zeng, Jie Lin

**Affiliations:** ^1^ Department of Intensive Medicine (Comprehensive Intensive Care Unit), The First Affiliated Hospital of Gannan Medical University, Jiangxi Provincial Clinical Key Specialized Department of Intensive Medicine, Ganzhou, China; ^2^ Department of Medical Imaging, The First Affiliated Hospital of Gannan Medical University, Ganzhou, China; ^3^ Department of General Surgery (Hepatobiliary and Pancreatic Surgery), The First Affiliated Hospital of Gannan Medical University, Ganzhou, China

**Keywords:** fall from height injury, penetrating abdominal injury, fatal trauma, prognosis, multi-disciplinary treatment

## Abstract

Fall from height causing penetrating abdominal visceral injuries is rare condition leading to abdominal multiorgan damage. It carries high mortality. A case of construction site worker sustaining penetrating visceral injuries by falling from height leading to impalement of steal bar from anus presented to our hospital and managed by timely evacuation from site of accident to hospital, resuscitations, radiological investigations and multi-Disciplinary team management lead to successful outcome. Our protocol for such cases will be discussed with references.

## Introduction

Penetrating abdominal injury is a type of serious trauma that is extremely challenging in clinical work, often accompanied by multiple organ damage and dysfunction, massive bleeding, hemodynamic instability and even respiratory and circulatory failure (1). It is usually complicated, urgent, and critical, the treatment of patients with penetrating injuries is rugged and arduous. Due to the special anatomical structure of the human body, choosing the appropriate surgical method according to the patient's body injury will maximize the chance of successful rescue. This article describes an extremely rare case of a construction site worker sustaining penetrating visceral injuries by falling from height leading to impalement of steal bar from anus presented to our hospital, this patient was rescued by the multidisciplinary team of our hospital and had an excellent prognosis, which will guide the field of emergency and critical care medicine.

## Case presentation

**Figure 1:**
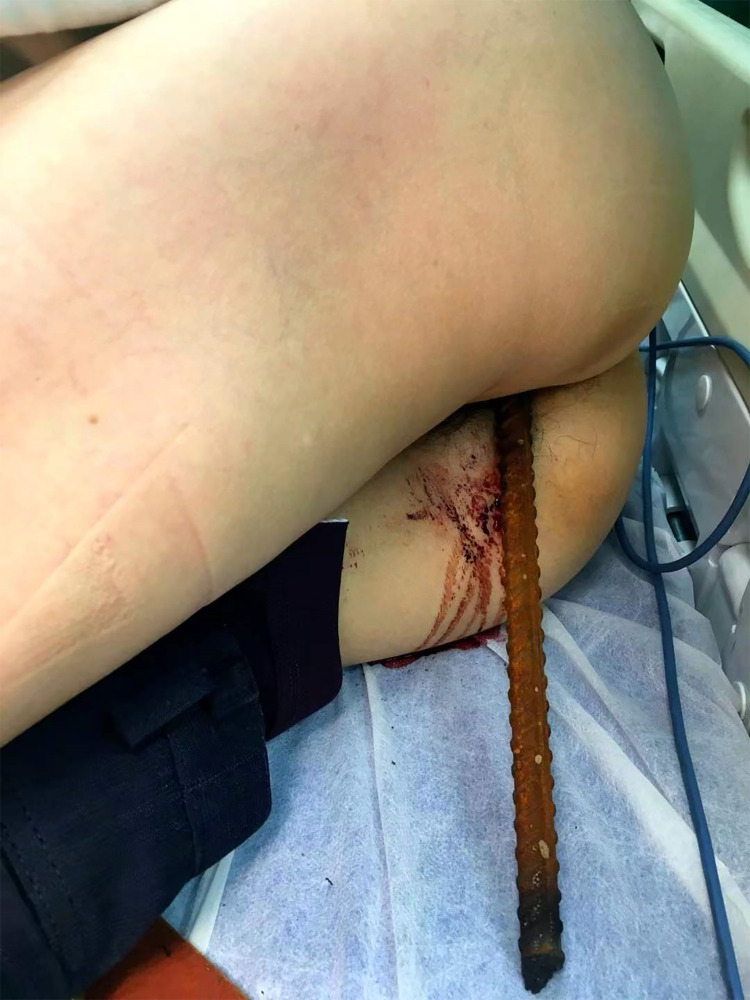
An inserted steel bar is visible at the anus.

A 59-year-old male patient who works as a construction worker was admitted to the First Affiliated Hospital of Gannan Medical University on the evening of February 27, 2021 with the main complaint of “falling from a height half an hour ago”. The patient accidentally fell from a height on the construction site half an hour before admission, and the steel bar was impaled from the anus. The patient was sent to the hospital by ambulance and then was transferred from the emergency department to intensive care unit (ICU). The patient was in poor mental state and physical activity was limited. Physical examination on admission showed body temperature (T): 36.5 °C, pulse (P): 112 beats/min, respiration (R): 20 beats/min, blood pressure (Bp): 123/80 mmHg, conscious, in the right lateral decubitus position, cardiopulmonary physical examination was unremarkable. It showed obvious abdominal tenderness and positive percussion pain in the right kidney area. An inserted steel bar is visible at the anus (Figure 1).

**Figure 2:**
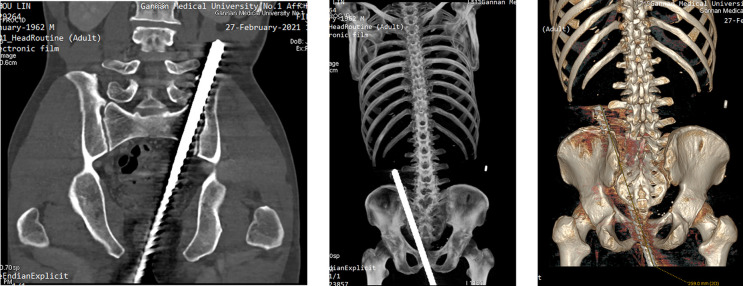
Abdominal CT scan + enhancement + post-processing (image reconstruction) showed that metal penetrating injury of left perineum, pelvis, left sacrum and ilium, the left internal iliac artery distal segment was not clearly displayed due to the artifact, and the relationship with the foreign matter was difficult to assess.

This patient was provided with a full abdominal CT scan + enhancement + post-processing (image reconstruction), the results of CT (Figure 2) showed that metal penetrating injury of left perineum, pelvis, left sacrum and ilium, the left internal iliac artery distal segment was not clearly displayed due to the artifact, and the relationship with the foreign matter was difficult to assess.

Investigations after admission lead to diagnosis of Penetrating abdominal injury, Digestive tract perforation? And may be other unknown injuries. The patient required multi disciplinary care in Intensive care unit. At 22:00 on February 27, 2021, laparotomy required to explore for urinary tract injury, vascular and nerve exploration and sacroiliac fracture open reduction under general anesthesia. The steel bar did not damage the artery, the foreign matter of the steel bar was gradually and slowly removed. Bladder and left ureter did not show any injury. Air inflation of rectosigmoid large bowel area required to find a 1.5 cm perforation was found at the junction, requiring cleaning the pelvis and bridge colostomy, the cystoscopy performed to rule out damage to bladder. The patient required Post operative care in ICU and mechanical ventilation for respiratory support monitoring. The first day after the operation, colostomy was working and intrabdominal drainage tube produced minimal fluid. The post operative diagnosis was revised to 1. Penetrating injury (perineal rectum-sacrum-iliac rebar penetration injury); 2. perforation of sigmoid colon; 3. Acute localized peritonitis. On March 1, 2021, the patient was extubated, stable near normal vital signs, transferred to the general ward. On March 2, the patient was instructed to have a liquid diet and strengthen nutrition, and the patient recovered and was discharged from our hospital on March 12, 2021. The closure of stoma was planned after undergoing CT scan of Pelvis and Abdomen, Barium Enema. Post operative period was uneventful. The living conditions and social activities of the patient were acceptable, and we were delighted to learn that he was able to perform some daily tasks.

## Discussion

Construction workers often suffer from penetrating injuries due to falling, mechanical collapse or other means originated from special working environment and lack of adequate protective equipment (2, 3). Penetrating injuries can be classified into two major categories, one is the body injury caused by the body striking a stationary object, and the other is the penetrating injury caused by the moving object penetrating the stationary body (4). The rare case presented in this article is the first type of penetrating abdominal injury caused by a fall from heights on the construction site onto ground rebar.

Cases of death and body damage caused by penetrating injuries are still rare in mainland China (5, 6). Since penetrating injuries are mostly caused by heterogeneous trauma mechanisms, penetrating injuries in European and American countries were mostly caused by violent crimes such as gunshot wounds or metal knife stabbing injuries; conversely, penetrating injuries caused by car accidents or industrial injuries were more common in the Asian population (7, 8). No matter what the cause of the injury is discussed, the physical damage caused by penetrating injury may be fatal and it is arduous to save the life of the injured person through clinical treatment or intervention.

Patients with penetrating injury were associated with a 3% increase in mortality for every additional minute of waiting in the emergency department without any treatment (9). In addition, the high mortality rate of penetrating injuries caused by various external factors is not only related to this severe type of trauma, but also to the treatment at the scene of the incident. The first-hand medical handling of the patients who suffered from penetrating injuries and the operation process during transportation may be accompanied by some wrong operations or rescue measures, which may aggravate the physical condition and lead to fatal and irreversible damage to these patients (10, 11).

Nevertheless, the penetrating abdominal injury is part of the trunk injury, it may caused progressive dyspnea, and are in critical condition and hemodynamically unstable. And it is more critical and safer to transfer to the operating room for surgery as soon as possible than to bluntly remove penetrating foreign bodies in the emergency department or rescue scene. After all, abdominal visceral injury is still an indication for emergency laparotomy (12). After the patient in this article was admitted to the hospital, we perfected the necessary examinations for him, we immediately organized a hospital-wide MDT expert discussion, and made a clinical decision in a short period of time. However, in critically penetrating patients with the “fatal triad” of hypothermia (<34 °C), acidosis (pH < 7.2), and coagulopathy, clinical emergency measures such as rapid rehydration, debridement, hemostasis, intra-abdominal packing, and avoiding further contamination and spread should be performed as soon as possible, and the above-mentioned emergency treatment measures should be urgent (13).

The CT imaging reconstruction of abdominal organ injury is particularly critical in identifying abdominal organ and tissue damage, and also provides guidance for the repair of abdominal organ damage during surgery. Correspondingly, penetrating abdominal trauma often causes rupture of the gastrointestinal tract, intra-abdominal infection is inevitable due to the abundant blood flow in the abdominal cavity and the use of antibiotics and thorough debridement are especially necessary. It is worth noting that penetrating trauma of the abdominal cavity is caused by external force, it often causes muscle and fascia injury and dysfunction, and then abdominal hernia occurs (14), although it is extremely rare, it should also be noted that this rare of late complications. Besides, non-surgical conservative management is not unwise for patients with penetrating abdominal trauma, provided that there is no major organ damage and risk of infection or hemorrhagic shock, whereas the presence of peritonitis and the need for resection of abdominal organ damage are indications for emergency surgery (exploratory laparotomy) when hemodynamic instability (15, 16). Whether antibiotics should be used prophylactically before or after surgery is still controversial for patients with penetrating abdominal trauma, the benefit or harm of prophylactic use of antibiotics in reducing complications such as sepsis, intra-abdominal abscess, and wound infection needs to be considered by clinicians which based on clinical and evidence-based medical evidence (17).

We describe an extremely rare case of penetrating abdominal trauma caused by a fall from a height, interestingly, the steel bar ingeniously bypassed the large blood vessels, parenchymal organs and nerves, and vertebral bodies of the abdominal cavity, and only caused the rupture and perforation of the sigmoid colon. In addition, the patient underwent surgery within a short period of time after injury, which prevented the spread of infection. Perhaps the above-mentioned “coincidences” are what led to the miraculously good prognosis of this patient. It is meaningful to create an emergency green path for patients with penetrating abdominal injuries, it saves the time and process of rescue for those patients, thereby saving the lives of the patients. Correspondingly, the evaluation and rescue in the rescue team is also particularly critical, each member of the rescue team plays an paramount role in the pain management, psychological counseling, and doctor-patient communication (18).

## Conclusion

Penetrating abdominal trauma is extremely rare in clinical work, and is usually difficult to treat and has a high mortality rate. A multidisciplinary team including emergency medicine, critical care medicine, internal medicine and surgery has played an indispensable role in providing rescue procedures and smooth and timely diagnosis and treatment procedures for the patients with penetrating abdominal injuries. It greatly improves the quality and speed of rescue, which is extremely important for improving patient outcomes. Just like the patient reported in this paper, he recovered well and had a good prognosis without any complications after various professional treatments. In short, in addition to providing a safe and potentially low-risk working environment for construction workers, training a qualified first aid team and forming a professional rescue team are also critical. The case sharing in this article is expected to bring new guidance and thinking to the medical practice of penetrating abdominal trauma, a rare but fatal condition.

## Data Availability

The original contributions presented in the study are included in the article/Supplementary Material, further inquiries can be directed to the corresponding author/s.
